# Comparison of the neutrophil-to-lymphocyte and platelet-to-lymphocyte ratios in normoglycemic and hyperglycemic subjects

**DOI:** 10.31744/einstein_journal/2019AO4403

**Published:** 2018-12-28

**Authors:** Brena Barros Mendes, Anniely Carvalho Rebouças Oliveira, Keila Correia de Alcântara

**Affiliations:** 1 Hospital das Clínicas, Universidade Federal de Goiás, Goiânia, GO, Brazil; 2 Faculdade de Farmácia, Universidade Federal de Goiás, Goiânia, GO, Brazil

**Keywords:** Type 2 *diabetes mellitus*, Neutrophils, Lymphocytes, Blood platelets, Inflammation, Glycated hemoglobin A, *Diabetes mellitus* tipo 2, Neutrófilos, Linfócitos, Plaquetas, Inflamação, Hemoglobina A glicada

## Abstract

**Objective:**

To compare the neutrophil-to-lymphocyte and platelet-to-lymphocyte ratios as possible parameters of systemic inflammation in hyperglycemic and normoglycemic subjects.

**Methods:**

A retrospective, cross-sectional study of data collected from patients tested for fasting blood glucose, glycated hemoglobin (HbA1c) and blood count on the same day, between July and December 2016. Patients were divided into hyperglycemic and normoglycemic, and matched by age and sex. The data were analyzed using Epi Info™, version 7.2.1.0, for the Windows^®^ platform.

**Results:**

We enrolled 278 subjects, 139 hyperglycemic and 139 normoglycemic. The absolute number of leukocytes and neutrophils was higher in the Hyperglycemic Group (p=0.006 and p=0.004, respectively). There was no difference in the neutrophil-to-lymphocyte ratio between the Hyperglycemic Group and the Normoglycemic Group (2.1 *versus* 2.0; p=0.264), and both neutrophil-to-lymphocyte and platelet-to-lymphocyte ratios showed no differences between those with HbA1c ≥7% (n=127, p=0.778) and those with HbA1c <7% (n=12, p=0.490). In contrast, the platelet-to-lymphocyte ratio was lower in the Hyperglycemic Group (117.8 *versus* 129.6; p=0.007).

**Conclusion:**

Hyperglycemic subjects had a neutrophil-to-lymphocyte ratio similar to that of normoglycemic subjects, but had a lower platelet-to-lymphocyte ratio. Future prospective studies will be useful to determine the importance and prognostic value of neutrophil-to-lymphocyte and platelet-to-lymphocyte ratios in the hyperglycemic state.

## RESUMO

**Objetivo:**

Comparar a razão neutrófilo-linfócito e a razão plaqueta-linfócito como possíveis parâmetros de inflamação sistêmica em indivíduos hiperglicêmicos e normoglicêmicos.

**Métodos:**

Estudo transversal retrospectivo, em que foram coletados dados dos pacientes que realizaram glicemia em jejum, hemoglobina glicada (HbA1c) e hemograma na mesma data, entre julho e dezembro de 2016. Os pacientes foram divididos em um Grupo Hiperglicêmico e um Grupo Normoglicêmico, pareados por idade e sexo. Os dados foram analisados no Epi Info™, versão 7.2.1.0, em plataforma Windows^®^.

**Resultados:**

Foram incluídos 278 indivíduos, sendo 139 hiperglicêmicos e 139 normoglicêmicos. O número absoluto de leucócitos e neutrófilos foi maior no Grupo Hiperglicêmico (p=0,006 e p=0,004, respectivamente). Não houve diferença da razão neutrófilo-linfócito entre o Grupo Hiperglicêmico e o Grupo Normoglicêmico (2,1 *versus* 2,0; p=0,264), assim como a razão neutrófilo-linfócito e razão plaqueta-linfócito não apresentou diferença entre aqueles com HbA1c ≥7% (n=127) e com HbA1c <7% (n=12; p=0,778 e p=0,490). Contrariamente, a razão plaqueta-linfócito mostrou-se menor no Grupo Hiperglicêmico (117,8 *versus* 129,6; p=0,007).

**Conclusão:**

Indivíduos hiperglicêmicos apresentaram razão neutrófilo-linfócito semelhante a dos normoglicêmicos, mas tiveram razão plaqueta-linfócito menor. Pesquisas futuras, de modo prospectivo, seriam úteis para analisar a importância e o valor prognóstico da razão neutrófilo-linfócito e da razão plaqueta-linfócito no estado hiperglicêmico.

## INTRODUCTION

Prolonged hyperglycemia promotes the development of different complications resulting from inflammation, such as neuropathy, retinopathy, renal failure, hypercoagulability, hypertension, myocardial infarction, stroke, and peripheral vascular disease, among others. High blood glucose levels are toxic to the body, leading to protein glycation, hyperosmolality and increased intracellular sorbitol levels.^(^ ^1^ ^,^ ^2^ ^)^

Glycation is an irreversible non-enzymatic binding reaction between glucose and a protein – *e.g.* hemoglobin –, which gave rise to the term glycated hemoglobin (HbA1c). Hemoglobin A (HbA) is the main hemoglobin, with subtypes A1 and A2. Total HbA1 corresponds to the molecules most negatively charged by the addition of glucose and other carbohydrates. The A1c fraction is present in HbA1c, with glucose attached to the N-terminal valine of the beta chain. In addition to blood glucose, HbA1C has become an important tool for diagnosing *diabetes mellitus* (DM), ensuring glycemic control and predicting the risk of vascular complications.^(^ ^3^ ^)^

Inflammation is closely related with insulin-resistance, due to excess fat tissue producing pro-inflammatory adipokines, which results in low-grade chronic inflammation, impairing tissue response to insulin and leading to type 2 diabetes (T2DM).^(^ ^4^ ^,^ ^5^ ^)^ HbA1c levels >7% are associated with a higher risk of irreversible, organic injuries, but HbA1c is not predictive of inflammatory processes.^(^ ^1^ ^,^ ^6^ ^)^

In recent years, the neutrophil-to-lymphocyte ratio (NLR) and the platelet-to-lymphocyte ratio (PLR), which derive from a calculation of blood count parameters, were introduced as potential markers of inflammation in cardiac diseases, neoplasms and diabetes-associated complications.^(^ ^7^ ^–^ ^10^ ^)^

There are few studies in Brazil about the NLR and PLR in hyperglycemic states. Knowing that prolonged hyperglycemia results in severe complications and high morbidity and mortality, it is imperative that further studies be conducted to help determine the prognosis of chronic complications of T2DM.^(^ ^11^ ^,^ ^12^ ^)^ The goal is to potentially use blood count parameters, such as the NLR and PLR, as prognostic factors of inflammation, to minimize the incidence of complications in patients diagnosed with T2DM, considering how important inflammation is for the onset and progression of the disease.^(^ ^13^ ^,^ ^14^ ^)^

## OBJECTIVE

To compare the neutrophil-to-lymphocyte and platelet-to-lymphocyte ratios of hyperglycemic and normoglycemic subjects.

## METHODS

A cross-sectional, retrospective study looking at laboratory test results archived in the Laudos^®^ software, version 1.0.220, for patients seen in the Clinical Laboratory of the *Hospital das Clínicas da Universidade Federal de Goiás*, between July and December 2016.

To collect the data from the study population, we selected all patients aged ≥40 years tested for fasting glucose, HbA1c and complete blood count on the same day at the Clinical Laboratory, coming up to a total of 1,027 subjects within the established period.

We excluded patients with hematologic diseases, chronic kidney disease, pregnant women, subjects with acute infection, as described in the clinical indications field of the patient's registration form, and/or those with abnormal results in other laboratory tests. Moreover, in compliance with the 2015-2016 criteria of the *Sociedade Brasileira de Diabetes* (SBD) (https://www.diabetes.org.br/profissionais/images/docs/DIRETRIZES-SBD-2015-2016.pdf), and of the American Diabetes Association (ADA) (2014)^(^ ^6^ ^)^ for DM diagnosis, pre-diabetic patients with fasting glucose levels between 100 and 125mg/dL were excluded.

The selected patients (n=278) were divided into a Hyperglycemic Group with fasting glucose ≥126mg/dL and HbA1c ≥6.5%, and a Normoglycemic Group, with fasting glucose <100mg/dL and HbA1c <6.5%, matched by age and sex, according to the ADA.^(^ ^6^ ^)^ The Hyperglycemic Group was stratified into one group with HbA1c <7.0% and another with HbA1c ≥7.0%.

The hematologic evaluation, we collected, for all groups, the following complete blood count parameters: count of leukocytes (WBC), total neutrophils, lymphocytes and platelets. The NLR was calculated by dividing the total number of neutrophils by the number of lymphocytes, and the PLR, by dividing the number of platelets by the number of lymphocytes. Blood counts were performed using the Pentra DF Nexus blood analyzer (Horiba Medical, Montpellier, France). Blood glucose and HbA1c tests were performed on the Architect c4000 analyzer (Abbott Laboratories, Illinois, USA).

The study was approved by the Research Ethics Committee under number 855.670, CAAE: 21377413.7. 0000.5078 of October 29, 2014.

### Statistical analysis

For all groups, we calculated the median, maximum and minimum values of the variables age, sex, blood glucose levels, HbA1c, WBC count, total neutrophils, lymphocytes, platelets, NLR and PLR. The comparison was made using the Kruskal-Wallis nonparametric test and the χ^2^ test (only for the variable sex), and significance was established at p<0.05. The data were analyzed on the Epi Info™ software, version 7.2.1.0, for Windows^®^.

## RESULTS

Of the 1,027 subjects selected, 278 were enrolled in the study, 139 of which were hyperglycemic and 139, normoglycemic. Of this total, 71% (198/278) were female, 54% (149/278) were aged over 60 years, and 99.3% (276/278) were outpatients (Table 1). The NLR was similar between the Hyperglycemic Group (2.1; 0.5 to 12.1) and the Normoglycemic Group (2.0; 0.8 to 6.2) p=0.264, while the PLR was lower in the Hyperglycemic Group (117.8; 36.4 to 296.1) when compared to the Normoglycemic Group (129.6; 52.6 to 269.1), p=0.007.

**Table 1:** Demographic and laboratory parameters of the hyperglycemic and the Normoglycemic Groups

Parameters	Hyperglycemic Group (n=139)	Normoglycemic Group (n=139)	p value
Age*	62 (40-92)	63 (40-96)	0.761
Sex, F/M^†^	99/40	99/40	0.500
Fasting glucose, mg/dL*	190 (129-568)	91 (60-99)	<0,001
HbA1c, %*	8.5 (6.5-15.3)	5.5 (4.7-6.4)	<0.001
Total leukocytes, mm³*	6,600 (3,600-15,700)	6,200 (4,400-10,700)	0.006
Total neutrophils, mm³*	4,032 (1,620-11,932)	3,550 (1,927-7,383)	0.004
Lymphocytes, mm³*	1,960 (567-5,324)	1,825 (1,000-3,885)	0.213
Platelets, mm³*	232,000 (64,000-428,000)	238,000 (150,000-399,000)	0.270
NLR*	2.1 (0.5-12.1)	2.0 (0.8-6.2)	0.264
PLR*	117.8 (36.4-296.1)	129.6 (52.6-269.8)	0.007

Results expressed as medians (minimum-maximum).

* Kruskal-Wallis non-parametric test;

† χ^2^ test. F: female; M: male; HbA1c: glycated hemoglobin; NLR: neutrophil-to-lymphocyte ratio; PLR: platelet-to-lymphocyte ratio.

The median NLR for the group with HbA1c <7% (n=12) was 2.0 (0.9 to 8.8) and for the group with HbA1c ≥7% (n=127), 2.1 (0.5 to 12.1), p=0.778. The PLR for the group with HbA1c <7% was 106.4 (72.6 to 269.5) and for the Decompensated Group with HbA1c ≥7%, 117.9 (36.4 to 296.1), p=0.490 (Table 2).

**Table 2:** Demographic and laboratory parameters of the Hyperglycemic Group, according to glycated hemoglobin levels (HbA1c)

Parameters	HbA1c <7% (n=12)	HbA1c ≥7% (n=127)	p value
Age*	65 (52-83)	61 (40-92)	0.042
Sex, F/M^†^	6/6	93/34	0.080
Fasting glucose, mg/dL*	152 (129-257)	194 (129-568)	0.007
Total leucocytes, mm³*	7,150 (3,600-12,100)	6,600 (3,600-15,700)	0.344
Total neutrophils, mm³*	4,218 (1,620-9,163)	4,032 (1,804-11,932)	0.435
Lymphocytes, mm³*	2,061 (909-3,388)	1,955 (5.67-5,324)	0.997
Platelets, mm³*	251,000 (89,000-308,000)	230,000 (64,000-428,000)	0.848
NLR*	2.0 (0.9-8.8)	2.1 (0.5-12.1)	0.778
PLR*	106.4 (72.6-269.5)	117.9 (36.4-296.1)	0.490

Results expressed as median (minimum-maximum).

* Kruskal-Wallis non-parametric test;

† χ^2^ test. F: female; M: male; NLR: neutrophil-to-lymphocyte ratio; PLR: platelet-to-lymphocyte ratio.

## DISCUSSION

Some studies showed chronic inflammation plays an important role in the development and progression of T2DM.^(^ ^15^ ^–^ ^17^ ^)^ The PLR and the NLR have been recently used as markers of systemic inflammation in chronic diseases, as well as prognostic predictors in cardiovascular diseases and neoplasms.^(^ ^18^ ^,^ ^19^ ^)^ The present study showed a lower PLR in hyperglycemic subjects, and found no difference in the NLR, when comparing hyperglycemic and normoglycemic subjects.

This lower PLR may be related with increased activation and platelet aggregation in patients with T2DM. This is attributable to deficient secretion of insulin, which has the role of inhibiting platelet aggregation, oxidative stress and endothelial damage from glucotoxicity. Lower platelet counts in diabetic patients may be due to the decreased survival and/or production, and increased turnover of platelets.^(^ ^20^ ^)^

Despite their NLR not being higher than that of the Normoglycemic Group, the Hyperglycemic Group had higher total leukocytes and neutrophils than controls, in line with the results from other studies.^(^ ^10^ ^,^ ^21^ ^)^ Chronic inflammation in T2DM progresses with leukocyte recruitment to the vascular environment in response to oxidative stress and production of proinflammatory cytokines.^(^ ^22^ ^)^ The results of Vozarova et al. showed that a high WBC count is a predictive factor for the development of T2DM complications.^(^ ^23^ ^)^ Thus, low-grade chronic inflammation may play an important role in the progression to T2DM, as well as in increasing the morbidity and mortality of DM patients.^(^ ^2^ ^,^ ^24^ ^)^

The PLR and, particularly, the NLR have been associated with microvascular and macrovascular complications in diabetes, most importantly in disease progression and metabolic impairment.^(^ ^25^ ^–^ ^28^ ^)^ In a study by Sefil et al., NLR correlated positively with HbA1c levels.^(^ ^29^ ^)^ This study showed no difference between the ratios of hyperglycemic subjects with HbA1c <7.0% and hyperglycemic subjects with HbA1c ≥7.0%. Some factors, such as disease duration, use of drugs and other clinical conditions may have contributed to our findings.

Despite some limitations, there are few studies in Brazil correlating NLR, PLR and hyperglycemic states. That is why it is important to study these ratios, considering that, in Brazil, the prevalence of T2DM is high, leading to a high demand for healthcare and financial resources, and the method for measuring NLR and PLR is low-cost and easily accessible.

## CONCLUSION

The platelet-to-lymphocyte ratio found in hyperglycemic patients was significantly lower than the platelet-to-lymphocyte ratio in normoglycemic patients, while the neutrophil-to-lymphocyte ratio did not differ between the groups.
